# Solution-focused intervention for sick listed employees with psychological problems or muscle skeletal pain: a randomised controlled trial [ISRCTN39140363]

**DOI:** 10.1186/1471-2458-6-69

**Published:** 2006-03-16

**Authors:** Pal Nystuen, Kare B Hagen

**Affiliations:** 1 Psykologbistand as, Kristian Augusts gate 13, 0164 Oslo, Norway; 2 National Resource Centre for Rehabilitation in Rheumatology, Diakonhjemmet Hospital, PO box 23 Vindern, 0319 Oslo, Norway

## Abstract

**Background:**

Long-term sick leave has been of concern to politicians and decision-makers in Norway for several years. In the current study we assess the efficacy of a solution-focused follow-up for sick-listed employees.

**Methods:**

Employees on long-term sick leave due to psychological problems or muscle skeletal pain (n = 703) were invited to participate in the project. Following self-recruitment, 103 were randomly allocated to receive solution-focused follow-up (n = 53) or "treatment as usual" (n = 50). The intervention was integrated within the regular follow up of six social security offices and organised as eight weekly solution focused work sessions. Effectiveness was measured by rate of return to work and health related quality of life (SF-36).

**Results:**

Intention to treat analysis showed no significant differences between the two groups for any of the outcome measures. Secondary analysis, comparing those who attended at least 50% of the sessions with the control group revealed a significant difference in favour of the active intervention group in the SF-36 subscale of mental health (Effect Size 0.56, p = 0.05). When comparing the subgroup of participants with psychological problems there was a significant difference in mental health in favour of the intervention group (Effect Size 0.71, p = 0.041).

**Conclusion:**

A voluntary solution-focused intervention offered by social-security offices is no more effective than regular follow up for employees on long-term sick leave due to psychological problems or muscle skeletal pain.

## Background

The global burden of mental health problems has been addressed by the World Health Organization as a major challenge for the next decades [1,2]. A recent publication points to the need to reallocate resources to meet the needs for treatment [3]. Sick listed employees with psychological problems and muscle skeletal pain make up 61% of absences in the Norwegian work force [4]. In a previous study, we reported the yearly incidence for long-term sick leave due to psychological problems as 2.47% [5]. This accounted for 16.8% of all absences and 31.5% of long-term sick leave days in Norway in 1997–98. The number of employees with mental health problems receiving disability pension increased by 115% from 1990 to 2000 [4].

One approach in dealing with increasing levels of sick leave is to work indirectly on a policy level, trying to change the organisational routines as well as the attitudes of the employers. An example of a large-scale approach in Norway is the social security arrangement called "Active Sick Leave" that was launched in the early 1990's making it possible for absentees to attend work by performing different and less strenuous tasks. An extensive evaluation of a strategy to increase the use of Active Sick Leave was not able to detect any effects on absence rates or improved health for back pain patients [6,7].

The medical model builds on the underlying assumption that finding the cause of the current problem and making the diagnosis will lead to a cure that will resolve the situation. This approach has proven very useful in detecting, diagnosing and curing illness. However, in more diffuse and complex situations involving psychological stress and pain, we argue that this approach might be of limited value. We believe a focus on health, possibilities and future goals could be an important supplement for many persons in this type of situation. Should we keep treating most cases of mood variations as diseases or would it be more appropriate to develop other modes of facing this challenge? Framing the situation as a personal "change project" as opposed to a more official and possibly stigmatising "illness project" can make a big difference.

Our project, called "Solutions at Work", aimed to develop and evaluate a solution-focused intervention for employees on long-term sick leave. In a previous RCT we investigated the feasibility and effectiveness of informing about and offering this intervention to sick listed employees with mental health problems or muscle skeletal diseases [8]. The information elements (invitation letter, telephone contact and invitation to an information meeting) were based at the local social security offices and integrated into their ordinary follow-up procedure of employees on long-term sick leave. While the letter was sent to all, only 31% were reached by telephone and 15% attended the information meetings. Only thirteen employees (11.5%) in the intervention group participated in the solution-focused follow-up, and we concluded that offering no difference was found in length of sick leave between the intervention and control groups. However, those who participated gave positive feed back regarding the course, and we therefore decided to evaluate the effects of participating in a solution-focused follow-up for sick listed employees reporting a need for this intervention.

The aim of the current study was to assess the efficacy of participating in a solution-focused intervention for sick listed employees with mental health problems or muscle skeletal disease. Efficacy was measured by the number of absence days per person, work status as well as perceived health status.

## Methods

Employees on long-term sick leave due to psychological problems or muscle skeletal pain (n = 703) were invited to participate in the project. One hundred and three accepted to participate, gave their informed consent and were included between February 2002 and February 2003.

### Design

The study was a randomised controlled trial of a solution-focused follow-up versus regular follow-up.

### Participants

Employees sick-listed for more than seven weeks due to non-severe psychological problems or muscle skeletal pain were eligible (table 1).

Table 1 about When Norwegian employees are absent from work for more than three consecutive days, they are required to consult a physician, usually a general practitioner (GP). Absentees are registered by their local social security office from the seventeenth day off work when the responsibility for sick leave benefits is passed from the employer to the welfare system. There is full wage compensation for twelve months. After seven to twelve weeks on sick leave a more thorough medical examination by the GP and a follow-up by the local security office is required. The participants in this study were included on the basis of the diagnosis made by the GP at seven or more weeks. The International Classification of Primary care (ICPC) has been used by Norwegian physicians since 1992 and has been evaluated to have acceptable quality as a basis for further analysis [9]. The main criteria for inclusion are all diagnoses indicating psychological distress or burnout, and different conditions of muscle skeletal pain (ICPC chapters A, L and P). Employees with more serious psychological diagnoses were excluded because many of them would probably need treatment by mental health specialists. Some of the muscle skeletal diagnoses were also considered ineligible for the intervention and excluded (Table 1). The sample size was estimated based on the ability to detect a difference in length of sick leave. We estimated a sample size of approximately 150 patients per group would be needed to detect a difference of 21 or more days (α = 0.05 with 80% power). Due to recruitment problems and time limitations we were not able to reach this number of included persons.

### Recruitment and allocation

A standardised procedure was developed to ensure that all the employees who met the inclusion criteria were informed, included and randomised. The participants were recruited from six social security offices located in Oslo.

**Figure 1:**
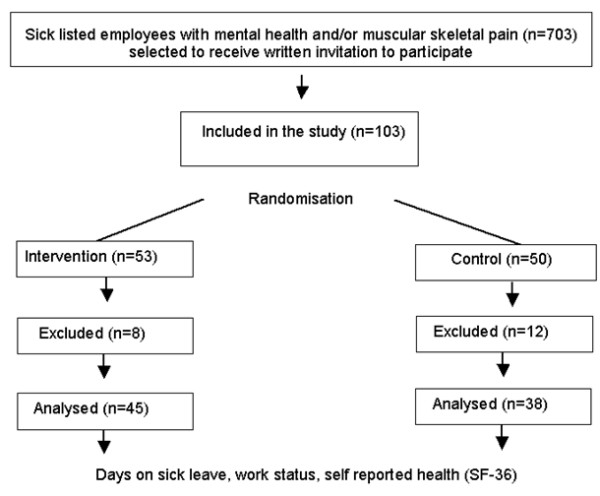
Flowchart of the trial.

As a part of the social security offices ordinary follow-up of employees on long-term sick leave (more than seven weeks), all employees with specified ICPC diagnoses were invited to take part in the project. A total of 703 persons were considered eligible and received a written invitation to take part in the study. Half of this population was randomly selected for attempted telephone follow up. We did this to increase the uptake as well as external validity of our study. Telephone follow-up increased uptake among non-responders significantly [10]. A total of 103 persons were included in the study and randomised to an intervention group (n = 53) or a control group (n = 50) (Figure 1). In order to keep the numbers of subjects in each group similar, we randomised participants by block randomisation using computer generated random numbers. A researcher not involved in the recruitment process (KBH) performed the random allocation. Concealment was ensured by implementing the allocation sequence to the project team by numbered sealed envelopes who assigned the participants to their groups.

### Intervention

Solution-focused practice has been applied and developed for the last 25 years [11]. The method evolved from a clinical practice in Milwaukee where several of the founders of the "Brief Family Therapy Centre" have contributed with essential writings on this method [12-15]. A recent review of controlled studies, covering a wide range of treatment settings with various outcome measures, show preliminary support for the efficacy of solution-focused brief therapy [16]. The review included one study of rehabilitation of orthopaedic patients where return to work was one of the outcome measures [17] and demonstrated a significant difference in return to work in favour the intervention group. Acknowledging and utilizing the experiences and resources of the client are among the basic elements in solution-focused therapy and counselling.

The intervention group was offered solution-focused follow-up, either individually or in a group depending on individual preferences. The majority chose to take part in a group, but about half chose to have individual counselling following the group intervention. Four psychologists delivered the intervention. They were familiar with solution-focused work as well as other therapeutic methods, in both individual consultations and group settings. The focus of the consultation and group work was the work situation, but any kind of topic was acceptable. Confidentiality was strictly observed and information was not shared with others, e.g. the employee's GP or employer, unless requested by the employee.

The intervention team developed what we called "The Road Ahead Course". The intervention was based on principles of solution focused group work [18-20]. The course consisted of eight weekly sessions of three to four hours where the main focus was on coping strategies, support between the participants and solutions and goals for the future. Half the time was spent in a plenary session where a topic of the day was introduced and discussed. These topics were: 1) Introduction 2) Self-esteem 3) Quality sick-leave 4) Communication 5) Conflict handling 6) Difficult choices 7) Coping with stress and 8) Follow-up. For example; in session six about "difficult choices", we used a version of a solution-focused interview involving questions to make the participants imagine a future where the problem was gone. The answers were followed up by probing for exceptions (whenever the problem did not happen) and scaling questions to make the participants more aware of their present status and the things that they need to do to make progress. These are essential elements of solution-focused work.

The other half of each session was spent in smaller groups of three to five participants where the work was organized according to solution-focused principles of goal description, support and constructive, specified feedback [12]. The therapists only supervised these groups. To guide the work in the small groups, we translated and adapted a concept of "reteaming", developed by Ben Furman and colleagues [21]. The participants were also invited to contact members of the "Solutions at Work" team if they felt a need for individual conversations at a later time.

The control group received "treatment as usual". This included a variety of treatments and activities that are variably available to persons in this situation, including psychotherapy and other treatment opportunities.

### Outcome measures

To assess the efficacy of the intervention we compared the intervention and control group on the mean length of sick leave after 12 months. Work status and self assessment of health status six months after the intervention were measured by SF-36, version 2 [22]. For the outcome on work status we present the results in terms of active status (job, part or full time) versus passive status (sick leave, rehabilitation or disability pension). There were twenty-five late responders returning the questionnaire between 7–10 months after the intervention started. Since they were equally distributed between the two groups: eleven in the control group and fourteen were in the intervention group (Odds Ratio 1.03 95 CI 0.40–2.66, p = 0.94), it is unlikely that this may have affected the results. The general practitioners responsible for the sick leave note received general information about the project, but were not informed about individual patients and whether they participated in the project or not.

The distribution of SF-36 scores was normally distributed in most of the subscales in our sample (except Physical functioning and Role Physical). Since the non-parametrical test (Mann-Whitney U) showed no statistical differences in PF- and RP scores, only the parametric test results are shown in Table 5

### Data collection and analyses

Data on lost workdays, diagnosis, gender and age were obtained from the computerised registers of the National Insurance Agency twelve months after the participants concluded the intervention. All lost workdays and separate spells after inclusion in the project were recorded and added up for each individual. Work status was measured by the information in the NIA register as well as by a questionnaire. We collected work status information on all patients (n = 83). Data to determine differences in health related quality of life were collected by postal questionnaires at baseline and six months after inclusion. Health care use was assessed in the six months follow up questionnaire. Due to low response rate after one reminder at the six months follow up questionnaire, data from the non-responders were collected by telephone.

The primary analysis was an intention to treat analysis comparing all respondents on the basis of treatment allocation. Secondary analysis compared groups of participants with mental health problems and those with muscular skeletal disease separately. Further, secondary analyses compared those in the intervention group who had attended at least 50% (four times) of the work sessions with the control group.

Due to multiple comparisons in the statistical analysis, we considered using the Bonferroni correction to reduce the risk of type I error. However, since the primary analyses showed no effect and this method is highly conservative (in fact some p-values would have exceeded 1), we chose not to use this correction.

Traditional descriptive statistics were used. Difference in length of sick leave between the groups was tested on the basis of intention to treat analysis with Students t-test. Because the number of sick days may not be normally distributed we also employed non-parametric statistics (Mann Whitney) for this test. χ^2 ^tests were used for categorical data. Effect sizes were calculated for differences in the SF-36 scores and results graded according to Cohens classification [23]. The analysis was done on a personal computer with SPSS, version 11.0.

### Ethics

The Regional Medical Ethics Committee approved the project.

## Results

In total, 103 persons were included. Fifty-three were allocated to the intervention group and 50 to the control group (table 2). Twelve persons in the intervention group and eight in the control group were excluded due to: insufficient language skills (3 persons); wrongly offered the intervention while allocated to the control group (2 persons); wrong diagnosis (4 persons); received the offer after starting rehabilitation (2 persons); pregnancy (2 persons); less than 50% sick leave (5 persons); withdrew from the study (2 persons).

Baseline data on gender, age and perceived health indicate only small, insignificant differences between control and intervention group. For health related quality of life the response rate at baseline was 82% in the intervention group and 71% in the control group. After six months the response rate was 84% and 71% respectively.

### Absence days and work status

We measured days on sick leave after the intervention started. The length of sick leave and results of statistical parametric tests are shown in table 3. There were no differences in sick leave (table 3).

Six months after the intervention started 39.1% in the intervention group had returned to work compared to 27% in the control group (table 4), corresponding to an absolute risk difference at 12,1% or Number Needed to be Treated of eight. However, this difference was not statistically significant (p = 0.29).

### Health related quality of life (SF-36)

**Figure 2:**
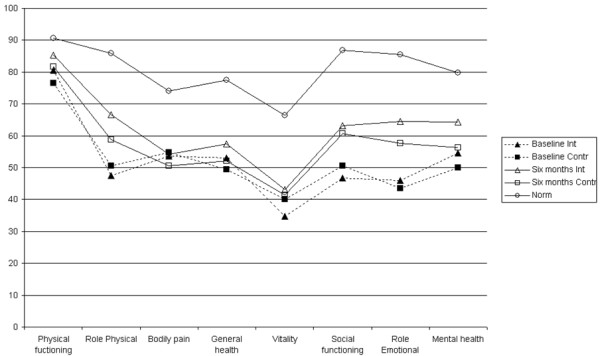
SF-36 scores.

Perceived health status was measured by the questionnaire SF-36 (table 5). The SF-36 profile is shown in Figure 2. No statistically significant differences were found between the groups. Greatest difference was found for the mental health sub scale (Effect Size 0.35, p = 0.17)

### Secondary analyses

As pre-specified in the research protocol we analysed sub-groups of participants. Interesting differences were found when comparing participants with mental health problems (n = 25) with control (n = 15) on the SF-36 subscales of social functioning (mean diff 15.5, p = 0.067, ES = 0.62) and mental health (mean diff 15.5, p = 0.041, ES = 0.71). Effect sizes for the other subscales varied from small (0.26) to moderate (0.48). Differences in lost workdays and status after six months were non-significant for both sub-groups.

When comparing those in the intervention group who attended at least four of the work sessions with the control group, a significant difference in favour of the intervention group was found in the mental health subscale (p = 0.05, ES = 0.56). For the other outcome measures there were no significant changes from the intention to treat analyses.

The follow up questionnaire also had questions on the use of health services. The intervention group had an average of 18.0 consultations with different health personnel, while the control group averaged 20.1 consultations. This difference was not statistically significant.

## Discussion

Our results show that a solution-focused follow-up for employees sick listed due to psychological problems or muscle skeletal pain is no more effective than regular follow up from the social security office. However, the observed differences in the subgroup on sick leave due to mental health problems are clinically relevant, and should be examined in a sufficiently powered trial.

The strength of this study is that it is a randomized comparison between the active intervention and regular follow up. This study was done in a practical setting thus increasing the external validity of the study, making generalization to an ordinary treatment setting less problematic.

The differences in the SF-36 mental health subscale and work status can be considered as practical relevant differences, although not statistically significant in the intention to treat analyses. In our research protocol we calculated an ideal sample size of 300. We ended up with 83 participants. Thus, the lack of statistical power makes it hard to draw any firm conclusions from this trial. However, the observed differences in mental health would have been statistically significant with a sample size of 120 in each group. Ideally we would have continued the project until reaching the needed number of patients, but the low uptake rate to the intervention and limitations of our project funding made this impossible.

We originally intended to include persons that had been sick listed for 7 to 12 weeks. To be able to recruit enough persons to fill our courses we ended up including absentees up to 12 months. This made the groups very heterogeneous in terms of absence rates as well as health status, making it more difficult to establish differences as statistically significant.

Performing a randomised controlled trial in close collaboration with local social security was an important goal of the project. A direct consequence of this was the high rate of post randomisation exclusions due to lack of screening procedures (see figure 1). We wanted to assess the effects of this intervention as it would work in a realistic setting.

Reducing the length and amount of sick leave is an important issue for both politicians as well as practitioners in Norway. However, few studies have reported any effective interventions in reducing sick leave. Typically one multi disciplinary intervention for patients on sick leave due to muscular pain reported significant effects on physical and psychological health, but found no significant differences on the return to work rate [24]. In a later study the same group of researchers described how patients with poor prognosis seemed to benefit from a multidisciplinary treatment program while it made no difference on return to work rates for patients with good prognosis [25]. This has important implications for the interpretations of our study. Our participants make out a very heterogeneous group making comparison difficult, especially when it comes to work status or return to work.

We only found one directly comparable study looking at the effects on solution-focused intervention in terms of lost workdays. A Swedish study found that participants receiving a similar solution focused intervention returned to work at a greater rate in the six months following the intervention. However, the differences in lost work days evened out after twelve months [26].

Why did we choose a solution-focused approach? There is limited evidence showing specific effects of different types of psychotherapies [27]. Some argue that "common factors" is the change engine of all approaches and that general and contextual factors are more important than specific factors [28]. We chose a solution-oriented approach partly because of the limited time perspective of our interventions. Our aim was to turn a negative circle of failing health into positive circles of change. We never had an explicit aim to make our participants go back to work as soon as possible. This would be contrary to the solution-focused principle that people know what is best for them in the current situation. Our role as therapists and moderators of change is to support and enhance their actions. If we were to do this study over again we certainly would have employed the recently developed and simplified process measurement tools developed by Lambert [29] and applied in clinical practice by Miller, Duncan and others at the Institute of Therapeutic Change [30].

The lottery concept of an experimental trial might seem odd to many participants, even those involved in medical research [31], and probably even more so when it comes to social welfare or mental health services. Doing a pragmatic controlled trial in this area made us aware of the many practical and ethical challenges this raises. What became increasingly important to the project team were ethical issues. The underlying assumption in an RCT is that the possible effects of interventions in the study are "unknown" to the researchers. Some claim that this is rarely the case even in medical research [32]. We found ourselves saying, for instance, "*We don't know if this intervention will be good or bad for you*". Most research teams have a pretty good idea, and probably even some evidence that it will be effective. Without this background knowledge, most projects would never be funded. This is also the case in this project, where there was some evidence for the efficacy of solution-focused brief therapy [16], but mostly with regard to changes in symptom levels, not lost work days. The ethics of randomisation will always be an important issue in an RCT, and even more so when we actively recruit more participants. The project team struggled with the randomisation procedure. Some participants were very happy to win, some were more or less indifferent (to lose/win) and a few were very disappointed when they ended up in the control group. Any differences between groups could be due to the positive effects of our intervention, but it could also be partly due to the negative effect of being disappointed in a difficult situation.

## Conclusion

The main conclusions from this study indicate that the intervention is no more effective than standard follow-up in either improving return to work or increasing perceived health. The observed differences in the subgroup on sick leave due to mental health problems are clinically relevant, and should be examined in a sufficiently powered trial. Large pragmatic trials are difficult to perform within this field, but are needed to establish better evidence for current policy and practice.

## Competing interests

This study was made possible due to funding from the Royal Ministry of Health and Social Affairs. The research was planned and performed independently of the Ministry and we declare no conflict in interests.

## Authors' contributions

Both authors participated in the design of the study. Both coordinated and supervised the study. PN drafted the main sections of the background, methods and discussion. KBH supervised the statistical analysis and methods section. Both authors read and approved the final manuscript.

## Pre-publication history

The pre-publication history for this paper can be accessed here:



## Figures and Tables

**Table 1:** Inclusion and exclusion criteria

**General inclusion criteria:**
Psychological problems (ICPC chapter P)
Symptoms of general exhaustion and burn-out (A01 and A04)
Muscoloskeletal pain (ICPC chapter L)

**The following ICPC-diagnosis were excluded:**

Psychological problems: P70-73, P77, P80, P98
Muscoloskeletal pain: L70, L71, L72-76, L77-79, L80-82
Additional causes for exclusion: self employed, pregnancy, graded sick leave of less than 50%, those awaiting elective orthopaedic surgery, those becoming 66 or more in the present year, persons in need of interpreter to communicate

**Table 2:** Characteristics of the participants at baseline

**Characteristics**	**Intervention group**	**Control Group**	**P-value**
Mean age (SD)	38.4 (SD 10,1)	36.8 (SD 10,3)	
Musculoskeletal pain	14 (51.9%)	13 (48.1%)	
Psycological problems	31 (51.9%)	25 (44.6%)	
Women	34 (75.6%)	29 (76.3%)	
SF-36			
Physical functioning	80.59 (SD 18.3)	76.52 (SD 20.1)	0.43
Role Physical	47.43 (SD 35.2)	50.54 (SD 32.3)	0.73
Bodily pain	53.69 (SD 29.3)	54.78 (SD 27.6)	0.88
General health	53.00 (SD 21.2)	49.52 (SD 23.6)	0.56
Vitality	34.71 (SD 19.2)	40.00 (SD 23.3)	0.31
Social functioning	46.79 (SD 27.7)	50.54 (SD 27.1)	0.61
Role emotional	45.95 (SD 28.1)	43.48 (SD 33.1)	0.76
Metal health	51.52 (SD 22.0)	50.09 (SD 21.2)	0.45

**Table 3:** Absence days after 12 months

	**Intervention**			**Control**			**Mean Diff.**	**95% CI**	**p-val.**
	
	n	Median	Mean	n	Median	Mean			
	
Lost work days total	41	60.0	87.0	34	67.0	90.7	-3.8	(-43.49 – 35.97)	0.85

**Table 4:** Work status after 6 months

	**Intervention**	**Control**	**Total**
Returned to work, full or part time	18 (39.1%)	9 (24.3%)	27 (32,5%)
Sick leave, continued rehabilitation or other	28 (60.9%)	28 (75.7%)	56 (67.5%)
Total	46 (100%)	37 (100%)	83 (100%)

**Table 5:** Self reported health (SF-36) after 6 months

	**Intervention**			**Control**			**Mean Diff.**	**95% CI**	**p-val**	ES*
	n	Median	Mean (SD)	n	Median	Mean (SD)				

Physical functioning	38	87.5	85.26 (15.5)	27	85.0	81.60 (14.9)	3.66	(-4.0 – 11.3)	0.34	0.24
Role Physical	38	75.0	66.61 (32.9)	27	62.5	58.80 (29.1)	7.82	(-8.0 – 23.6)	0.33	0.24
Bodily pain	38	51.0	54.16 (25.5)	27	42.0	50.48 (27.9)	3.68	(-9.7 – 17.0)	0.58	0.14
General health	38	52.0	57.39 (23.6)	27	55.0	52.19 (28.1)	5.21	(-7.6 – 18.1)	0.42	0.20
Vitality	38	45.0	43.16 (20.8)	27	40.0	41.48 (27.7)	1.68	(-10.3 – 13.7)	0.78	0.07
Social functioning	38	62.5	63.16 (25.1)	27	62.5	60.65 (28.7)	2.51	(10.9 – 18.9)	0.71	0.09
Role emotional	38	58.3	61.18 (31.1)	27	50.0	57.72 (30.1)	3.47	(-12.0 – 18.9)	0.66	0.10
Metal health	38	70.0	64.53 (22.0)	27	60.0	56.30 (25.6)	8.23	(-4.0 – 20.4)	0.17	0.35

*ES = Effect Size
